# Bringing Statistics Up to Speed with Data in Analysis of Lymphocyte Motility

**DOI:** 10.1371/journal.pone.0126333

**Published:** 2015-05-14

**Authors:** Kenneth Letendre, Emmanuel Donnadieu, Melanie E. Moses, Judy L. Cannon

**Affiliations:** 1 Department of Molecular Genetics and Microbiology, University of New Mexico School of Medicine, Albuquerque, NM, United States of America; 2 Department of Computer Science, University of New Mexico, Albuquerque, NM, United States of America; 3 Inserm, U1016, Institut Cochin, Université Paris Descartes, Sorbonne Paris Cité, Paris, France; 4 Department of Biology, University of New Mexico, Albuquerque, NM, United States of America; 5 Santa Fe Institute, Santa Fe, New Mexico, United States of America; Centrum Wiskunde & Informatica (CWI) & Netherlands Institute for Systems Biology, NETHERLANDS

## Abstract

Two-photon (2P) microscopy provides immunologists with 3D video of the movement of lymphocytes in vivo. Motility parameters extracted from these videos allow detailed analysis of lymphocyte motility in lymph nodes and peripheral tissues. However, standard parametric statistical analyses such as the Student’s t-test are often used incorrectly, and fail to take into account confounds introduced by the experimental methods, potentially leading to erroneous conclusions about T cell motility. Here, we compare the motility of WT T cell versus PKCθ-/-, CARMA1-/-, CCR7-/-, and PTX-treated T cells. We show that the fluorescent dyes used to label T cells have significant effects on T cell motility, and we demonstrate the use of factorial ANOVA as a statistical tool that can control for these effects. In addition, researchers often choose between the use of “cell-based” parameters by averaging multiple steps of a single cell over time (e.g. cell mean speed), or “step-based” parameters, in which all steps of a cell population (e.g. instantaneous speed) are grouped without regard for the cell track. Using mixed model ANOVA, we show that we can maintain cell-based analyses without losing the statistical power of step-based data. We find that as we use additional levels of statistical control, we can more accurately estimate the speed of T cells as they move in lymph nodes as well as measure the impact of individual signaling molecules on T cell motility. As there is increasing interest in using computational modeling to understand T cell behavior in *in vivo*, these quantitative measures not only give us a better determination of actual T cell movement, they may prove crucial for models to generate accurate predictions about T cell behavior.

## Introduction

Time-lapse two photon microscopy provides stunning video of the 3-dimensional movement and interaction of immune cells within living tissue [1–4]. This technology affords us an unprecedented view into the behavior of immune cells *in vivo*. An on-going question is how best to analyze quantitative data extracted from such videos in order to more accurately describe T cell motility and determine differences in behavior across different T cell populations.

There has been increasing interest in precise quantitative analysis of T cell motility to parameterize computational models of immune responses [5–8]. The movement of thousands of T cells can be captured in videos of two-photon images, and the speeds and turning angles of each T cell can be calculated. However, there are difficulties in obtaining unbiased analyses of *in vivo* motility data. While the speed of a cell during an observation seems obvious to calculate (by dividing the distance a cell has traveled by the time the cell has been tracked in the video; see [4,9,10]), two different approaches have been used to estimate both speed and turning angle. In a review, Beltman, Maree and De Boer [9] highlighted “cell-based” versus “step-based” analyses. The most commonly used method is cell-based analysis which calculates the mean or median cell speed or migration angle for each continuously tracked cell. These ‘cell-based parameters’ are the unit of analysis with each derived mean or median treated as one data point. On the other hand, step-based analysis pools all observations of individual cells across all time steps without regard to the identity of individual cells that produced those observations ('step-based parameters'). We show below that these analyses can generate very different estimates of cell speed and turning angle. Because researchers use cell speed and turning angle to estimate motile behavior and calculate “motility coefficients” to characterize the scanning capacity of T cells [11,12], using cell-based versus step-based approaches can significantly affect our understanding of lymphocyte motility.

There is an additional difficulty in analyzing *in vivo* T cell movement using two-photon microscopy due to the complexity of experimental designs. T cells must be visualized using fluorescent vital dyes or tagging by fluorescent proteins. Fluorescent cells are introduced into recipient animals, multiple fields within multiple tissues are visualized, and the process is repeated for reproducibility. Motility can be affected by environmental conditions such as oxygenation, blood flow, and temperature [13]. The fluorescent dyes commonly used to visualize T cells can also affect motility. Dyes are chosen to balance the need for fluorescence intensity against the potential phototoxicity [13]. To control for potential confounding effects of the dyes methodologically, researchers often repeat experiments with dyes inverted with respect to the experimental cell populations [1,14–16]. However, few studies report sample sizes or quantitative results for these repeated trials, specific dye effects have not been quantitatively detailed, nor have methods been proposed to verify that dye effects are appropriately controlled for so that they do not alter quantitative or qualitative conclusions from experiments.

Finally, a standard statistical treatment of T cell motility data has not been established. In order to detect effects of specific signaling pathways on T cell motility, researchers compare motility of WT and gene-deleted populations. While many studies have used the Student’s t-test to detect differences [14,16,17], some studies have also used non-parametric techniques to analyze motility of T cell populations [4,13,17]. To date, there has been no systematic discussion of what statistical test is appropriate for analyzing T cell motility parameters.

In our current study, we compare the motility of WT T cells versus PKCθ-/-, CARMA1-/-, and CCR7-/- T cells as well as PTX treated T cells to determine the role of individual molecules in regulating T cell motility. We analyze these data using the commonly used t-test and Mann-Whitney U test to demonstrate the differences between the results obtained by these tests and the techniques we recommend, as well as to illustrate how these tests are inappropriate. These tests are inappropriate in analysis of T cell motility data because of the dependence introduced into the data by the experimental design, which these tests cannot account for. Furthermore, parametric tests such as the t-test assume that the residuals of the analysis are normally distributed, an assumption that T cell motility data often fails to satisfy. Non-parametric tests make no assumptions about the distribution of data or the residuals of these analyses and are therefore more appropriate for data for which the assumption of normality is violated. We show for the first time a method to combine the biologically relevant cell-based analysis with the statistical power of step-based data. We formally show the precise effect of specific dyes on T cell motility, and demonstrate the use of factorial ANOVA to statistically control for previously unaccounted for dye effects. We also suggest alternatives to the typically used parametric Student’s t-test for obtaining non-parametric estimates and p-values, in analysis of non-normally distributed data such as T cell speeds and migration angles. Readily available statistical tools (we use JMP 10.0.0 http://www.jmp.com but many others are available) allowed us to generate more accurate estimate measures of T cell motility in vivo. These more accurate estimates enhance our ability to identify how molecular regulators modulate T cell motility within lymph nodes, and they also provide better data to parameterize computational models. As researchers in this field make large investments to obtain detailed data afforded by two-photon microscopy, greater rigor in statistical analysis combined with better reporting methods can increase the reliability and reproducibility of published findings, addressing recently highlighted problems with statistical analyses and reporting [18,19].

## Material and Methods

### Ethics statement

The protocol was approved by the IACUC at the University of New Mexico (protocol # 10–100487). The breeding and maintenance of mice used in this research conform to the principles outlined by the Animal Welfare Act of the National Institutes of Health. All efforts were made to minimize suffering with use of ketamine and xylazine when appropriate. Euthanasia was performed by isofluorane overdose.

### Mice

C57BL/6 mice, B6.PKCθ-/-, and B6.CCR7-/- mice were from Jackson Laboratories (Bar Harbor, ME). B6.CARMA1-/- animals were a gift from Dr. Marisa Alegre (University of Chicago). All mice were bred and/or maintained in a specific pathogen-free condition in barrier facilities (Albuquerque, NM).

### Two photon imaging of explanted lymph nodes

For the WT:PKCθ-/-; WT:CARMA1-/-, SuB and PTX experiments, T cells were purified by nylon wool exactly as previously described [20] and purified T cells labeled with either 1μM CFSE (Invitrogen) or 5 μM CMTMR (Invitrogen, Carlsbad, CA). Both WT and knockout or PTX-/suB- (from Sigma Aldrich, St. Louis, MO) treated T cells were labeled with both CFSE and CMTMR to account for dye effects. 5 to 10 x10^6^ labeled T cells were injected I.V. into recipient mice and inguinal lymph nodes were removed 15–18 hours later and imaged using two photon-imaging. For PTX experiments, T cells were treated at a concentration of 5 to 10 ng/mL cells/mL with PTX (SIGMA, P159-40UG) or suB (SIGMA, P7208-50UG) at 5.10^6^/mL for 10 minutes in DMEM 10% FCS following the protocol described by [21].

**Fig 1:**
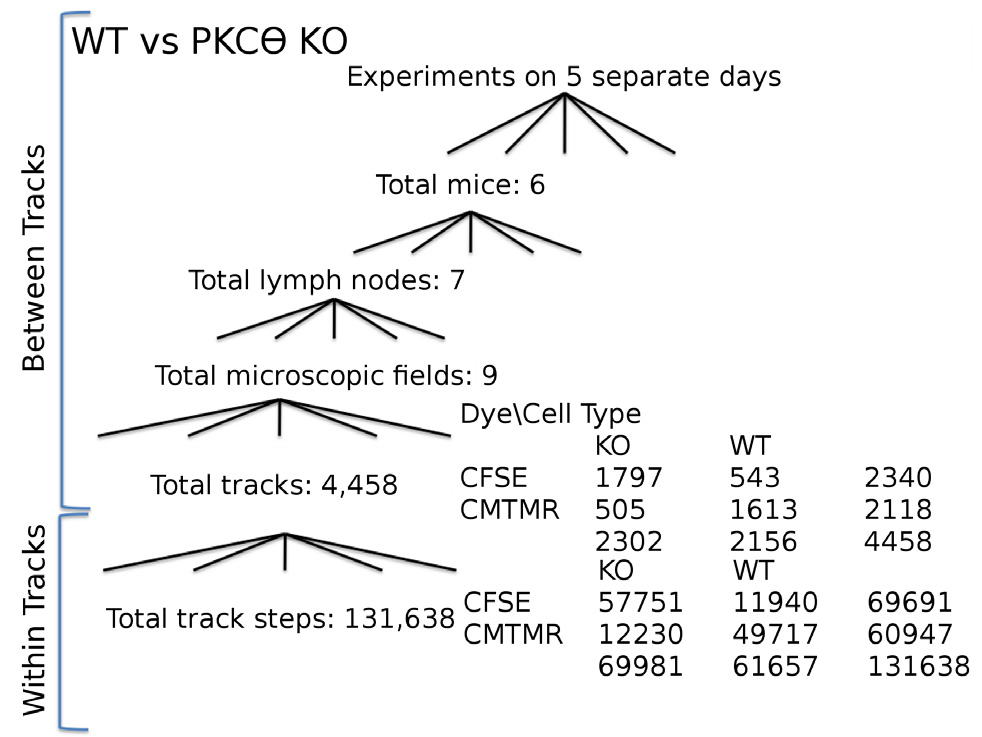
Experimental design and resulting data structure for WT vs PKCθ-/- experiments. Data on PKCθ-/- (KO) and wild-type (WT) T cell motility were collected during experiments on 5 days, using 6 total mice, from which 7 total lymph nodes were extracted, with observation in 9 total microscopic fields, in which 4,458 total tracks were observed, containing 131,638 total step observations.

WT:PKCθ-/- and WT:CCR7-/- imaging experiments were previously published. WT:PKCθ-/- experiments are described in Cannon et al., 2013; WT:CCR7-/- raw data (microscopy acquisitions) were taken from a published study [14] and reanalyzed. These experiments used cells dyed with 1 μM CMFDA or 2 μM fura-2 AM from Invitrogen (Carlsbad, CA) and imaging is described in [14]. The structure of the data is presented in Fig 1 and S1, S2 and S3 Figs.

WT:CARMA1-/- experiments were conducted with a 2-photon microscope in the Fluorescence Microscopy Facility in the UNM Cancer Center with a mode locked Ti:Sapphire infrared laser (Coherent Ultra II; tunable from 680–1080 nm; avg. power 3.5 W) for multiphoton fluorescence excitation. The microscope stand is a Zeiss Axiovert 200 with motorized XY stage and IR-corrected long working distance objectives (25X:multi-immersion and 40X:water immersion) and image acquisition via a Zeiss LSM510 scanhead. *Ex vivo* tissue and organs are maintained during microscopic observation in a stage microincubator system (LCI-Live Cell Imaging) equipped with heating, humidity, CO_2_ atmosphere and perfusion. Explanted lymph nodes were placed on a glass cover-slip in the chamber. The sample is perfused with a 37°C solution of DMEM (phenol red free, Gibco) bubbled with 95% O_2_ and 5% CO_2_. T cell behavior within a lymph node was monitored in the T cell area at a minimum of 50μm below the surface of the node. For 4D analysis of T cell motility, multiple stacks in the z axis (z step = 3μm) were acquired every 15–20sec (depending on the number of z stacks acquired) for 15–50 min, with an overall field thickness of 40–60μm. Cell motility was analyzed with Imaris software (version 6; Bitplane). Tracks that lasted fewer than 3 time steps (corresponding to tracks lasting less than 100 seconds using the duration filter in Imaris) were not taken into account in the analysis. Length filter (threshold of 17μm = 3 times the diameter of the cell), and displacement^2^ filter (threshold of 300μm^2^ = 17μm x 17μm) were also used to discard tracks of non-motile cells in the PKCθ and CARMA1 data sets. Videos were made by projecting the 4D information along the z axis in a single plane.

### Statistical methods

#### Student's t-test

The following simple equation describes the values of our data in two groups:

$$y_{ij}=\mu +\alpha_{i}+e_{ij}$$ (1)

where *y*_*ij*_ is each of our 1…*j* observations in each of our 1…*i* groups (*i* = 2 in this case; or if we were performing one-way ANOVA, *i* ≥ 3); μ is the overall mean; *μ*_*i*_ = *μ* + *α*_*i*_ is the mean for each group *i*; and *e*_*ij*_ is an error term for the variance left unexplained by our two groups (i.e. the residuals, or how much the value of each *y*_*ij*_ is above or below the group mean *μ*_*i*_). For the t-test, the assumption of normality requires that *e*_*ij*_ are distributed as *N*(0,*σ* ^2^) (according to a normal distribution with mean zero and variance equal to the sample variance *σ* ^2^ [22]). Eq (1) amounts to a model describing our observations *y* ^*ij*^ as the sum of the population mean *μ*_*1*_ or *μ*_*2*_ for our two populations of cells plus random deviation from that mean for each observation. We wish to know with what degree of confidence we can say that our population means *μ*_*1*_ ≠ *μ*_*2*_; a p-value gives us this confidence. (For more detail on the calculation of p-values see [22].)

#### Mann-Whitney U Test, and Non-Parametric Methods

The Mann-Whitney U test is a non-parametric counterpart to Student's t-test. In this test, observations are ordered from lowest to highest value, and assigned ranks from the first to the last value in this ordering, allowing estimates of differences in groups without regard to the magnitude of differences in values in the sample. Conover & Iman [23] have suggested rank transformation of data as a general technique to apply non-parametric tests using analyses designed for parametric statistics. Non-parametric tests such as Mann-Whitney U test and the Kruskal-Wallis test for differences in median, rather than mean, values as means are more heavily influenced by outliers in non-normal data. Basic formulas for the non-parametric methods are identical to those for the parametric methods. Calculations are performed on rank-transformed data.

#### Factorial ANOVA

We control for the effects of the fluorescent dyes on cell motility by including the dyes and their interactions with our cell populations as factors in factorial ANOVA. This allows us to examine the effects of different dyes on lymphocyte motility and simultaneously control these effects while estimating the effects of the cell populations of interest.

We can express the resulting model:

$$y_{ij}=\mu +\alpha_{i}+\beta_{j}+\alpha \beta_{ij}+e_{ij}$$ (2)

where *μ* is the overall mean; *α*_*i*_ is the effect of our cell populations; *β*_*j*_ is the main effect of the dyes; and *αβ*_*ij*_ is the interaction of the cell populations and the dyes [22]. We repeated this analysis using rank-transformed speeds and migration angles in order to obtain non-parametric estimates.

#### Mixed Modeling

To combine cell-based and step-based analyses, we used the track ID provided by Imaris as a predictor to control for individual cell behavior. Because differences amongst tracks within a WT or knockout population are not only factors we imposed experimentally, and because the cells we sampled are a sub-sample of all the cells we might have observed, we treated this as a nuisance variable ([22], p. 33) that we control by entering it into our model as a random effect variable, which mathematically incorporates the effect of track ID into the error term, rather than as a fixed effect of the model. Statistical control for track ID allows our model take into account the fact that individual cells may move differently than others, and also importantly, the fact that individual tracks have different durations, and thus different sample sizes of instantaneous steps. Our model, which now contains both fixed and random effects, is an example of mixed model ANOVA.

We add our variables containing unique IDs for each cell to Eq 2 giving:

$$y_{ijk}=\mu +\alpha_{i}+\beta_{j}+\alpha \beta_{ij}+\gamma_{k}+e_{ijk}$$ (3)

where *γ*_*k*_ is the random effect of individual cell behavior for each of our 1 … *k* observed cells, assuming that *γ*_*k*_ is independently distributed according to *N*(0,*σ*_*γ*_^*2*^) and that {*γ*_*k*_} is independent of {*e*_*ijk*_} [22]. We repeated this analysis using rank-transformed speeds and migration angles respectively in order to obtain non-parametric estimates.

#### Nested Factors

We enter date IDs, mouse, lymph node, and field, with the date as our top level random effect in this analysis. The other random effects we enter are nested within date, and within each other to match the hierarchical structure in our data (see Fig 1). We enter mouse IDs nested in date; lymph nodes nested in mouse and date; fields nested in lymph node, mouse, and date; and finally cells nested in field, lymph node, mouse and date. (See S5 Fig sfor model specification in JMP.) It is important to ensure that all the IDs at each level are unique (similar to our treatment of the cell IDs, our unique mouse IDs are concatenated strings of the date of observation and the mouse ID). Note that if there is perfect overlap between any of the nested levels, such as only one mouse used on each date of observation, the lower level variable will take up all the variance explained by the other level. This was the case in our CARMA1 dataset, for example, and we therefore excluded date from our nested analysis of these data.

We repeated this analysis using rank-transformed speeds and migration angles in order to obtain non-parametric estimates.

### Data and tool sharing

We have made our statistical analysis procedure available at the following website: http://stmc.health.unm.edu/tools-and-data/. The minimal dataset underlying this paper is also available at http://stmc.health.unm.edu/tools-and-data/index.html as comma-separated values (.csv) files which contain track data required to reproduce statistical analyses. Raw images files are available from the corresponding author upon request.

## Results

Our goal is to identify molecular regulators that modulate T cell motility within lymph nodes. Lack of CCR7 and pertussis toxin (PTX) inhibition of CCR7 and associated chemokine receptors have shown that CCR7 and chemokine receptor signaling is key to driving T cell motility within LNs [12,14,24]. We recently showed that PKCθ is activated downstream of CCR7 [20], and CARMA1 is an interacting partner of PKCθ [25]. To determine the effect of each signaling molecule on T cell motility, we compared the motility of WT T cells with T cells deficient in PKCθ, CARMA1, and CCR7 (data from [14,20]). We purified and visualized T cell motility using 2-photon microscopy of each population as described in the Methods. We calculated typical motility parameters such as speed and turning angles of individual cells in order to determine whether the lack of PKCθ, CARMA1, or CCR7 changed T cell motility. We also used PTX to inhibit T cell motility and used a non-active subunit B alone (suB) as the WT control.

**Fig 2:**
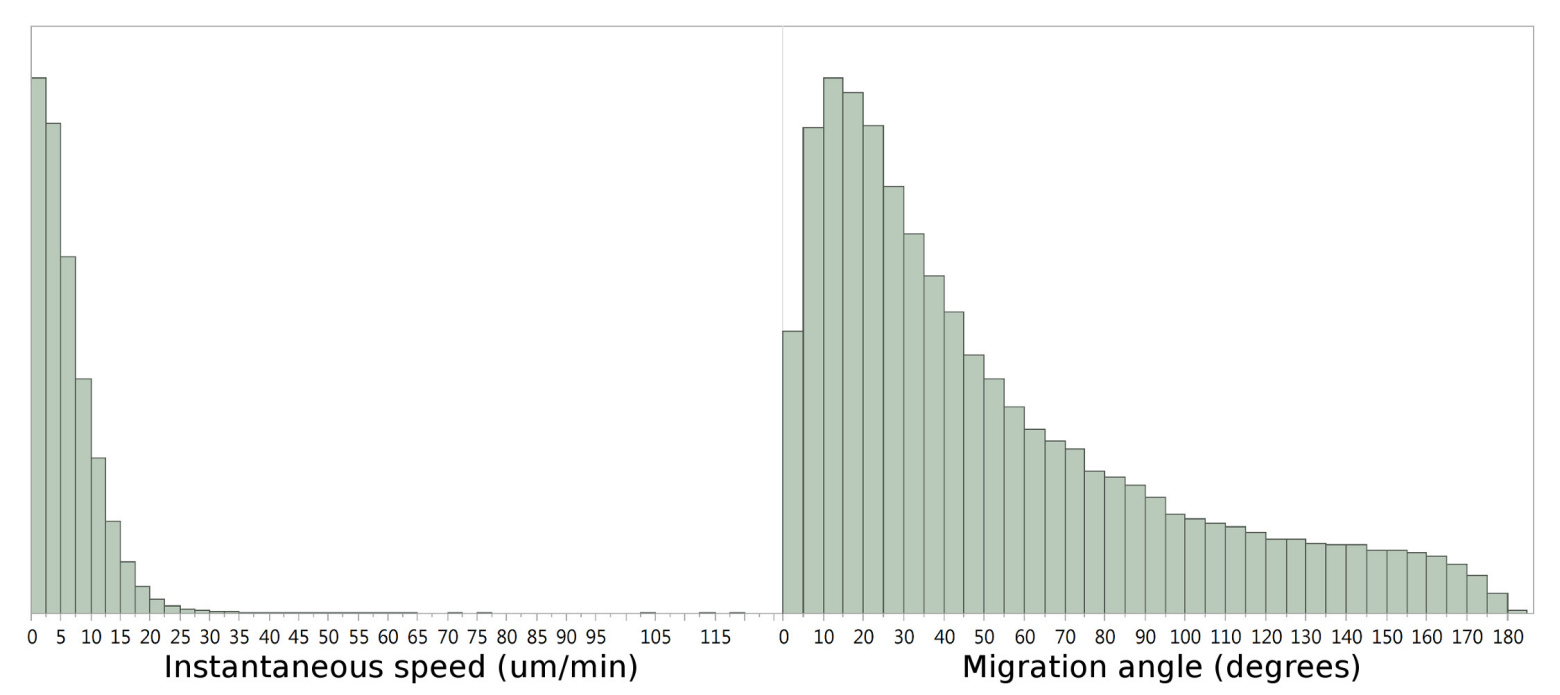
Distribution of step-based T cell speeds and migration angles in mouse lymph nodes. Histograms showing the distribution of motility parameters calculated from 2P microscopic observation of PKCθ-/- and wild-type T cells: A) speeds (in microns/minute) and B) migration angles (in degrees).

To formally assess whether parametric or non-parametric statistical tests should be used, we analyzed speed and turning angle distributions of WT T cells moving within LNs. T cell motility data—both migration speeds and angles, and particularly in step-based data—are not normally distributed, thus parametric tests like the t-test would be incorrect, requiring use of non-parametric statistical tests. Fig 2 and [1,26] show non normal distribution of instantaneous speed and turning angles; Fig 2 and [10] show non-normal distribution of instantaneous speeds. Thus, we performed the Mann-Whitney U test (for non-parametric data) for comparison to the Student's t-test (parametric data) on each of our four datasets: WT:PKCθ-/-; WT:CARMA1-/-; WT:CCR7-/-, and SuB:PTX. For each of the analyses we describe below, we repeat the analysis with rank-transformed data to obtain non-parametric estimates and p-values.

Using the Student’s t-test, we find that PKCθ-/-, CARMA1-/-, CCR7-/, and PTX treated T cells all showed statistically significant differences from WT T cell speeds (Table 1) as well as turning angles (Table 2). We find that the Mann-Whitney U test produced lower estimates for both speeds (Table 1) and migration angles (Table 2). This difference in estimates between the t-test and Mann-Whitney U test reflects the non-correspondence of the mean (t-test) and median (Mann-Whitney) due to the non-normality of these data. While the estimates of speed and turning angle are different, the magnitude of differences in the estimates (i.e. the effect sizes) as well as significance are generally similar with the Mann-Whitney U test compared to those obtained by Student's t-test.

**Table 1:** Analyses of track instantaneous speeds.

Analysis	Statistic	WT:PKCƟ-/-	WT:CARMA1-/-	WT:CCR7-/-	SuB:PTX
t-test	WT est.	6.48	8.61	5.16	6.54
	KO est.	6.34	8.28	4.16	4.59
	p-value	p<.0001	p<.0001	p<.0001	p<.0001
Mann-Whitney U test	WT est.	5.28	7.8	4.26	5.20
	KO est.	5.19	7.2	3.54	3.98
	p-value	p<.0001	p<.0001	p<.0001	p<.0001
Factorial ANOVA	WT est.	6.21	8.57	5.45	6.66
	KO est.	5.96	8.78	4.17	4.53
	p-value	p<.0001	p<.0001	p<.0001	p<.0001
Factorial ANOVA, rank-transformed data	WT est.	5.05	7.8	4.53	5.26
	KO est.	4.85	7.8	3.55	3.94
	p-value	p<.0001	p<.0001	p<.0001	p<.0001
Mixed model ANOVA controlling for cell IDs	WT est.	6.95	9.62	5.88	7.65
	KO est.	6.63	9.93	4.99	4.85
	p-value	p = 0.0021	p = 0.0035	p<.0001	p<.0001
Mixed model ANOVA, rank-transformed data	WT est.	5.58	8.4	4.91	5.94
	KO est.	5.34	9.0	4.14	4.16
	p-value	p = 0.0004	p = 0.0156	p<.0001	p<.0001
Nested ANOVA controlling for all experimental blocks	WT est.	7.28	9.02	5.89	7.22
	KO est.	6.84	9.29	5.00	4.63
	p-value	p<.0001	p = 0.0086	p<.0001	p<.0001
Nested ANOVA, rank-transformed data	WT est.	5.80	7.8	4.92	5.63
	KO est.	5.47	8.4	4.15	4.81
	p-value	p<.0001	p = 0.0304	p<.0001	p<.0001

Cell speed for wild-type (WT) vs. PKCƟ-/-, CARMA1-/-, and CCR7-/-, and suB (control) vs. PTX-treated T cells. All speed estimates are in μm/min.

**Table 2:** Analyses of track migration angles.

Analysis	Statistic	WT:PKCƟ-/-	WT:CARMA1-/-	WT:CCR7-/-	SuB:PTX
t-test	WT est.	51.9	40.3	47.3	46.5
	KO est.	52.9	39.2	54.0	49.3
	p-value	p<.0001	p<.0001	p<.0001	p<.0001
Mann-Whitney U test	WT est.	37.7	30.1	35.9	37.4
	KO est.	38.2	29.8	40.1	35.1
	p-value	p = 0.0058	p = 0.0021	p<.0001	p<.0001
Factorial ANOVA	WT est.	54.0	40.6	46.3	45.2
	KO est.	54.3	38.2	54.0	50.3
	p-value	p = 0.3872	p<.0001	p<.0001	p<.0001
Factorial ANOVA, rank-transformed data	WT est.	38.5	30.2	35.6	34.3
	KO est.	38.9	29.3	40.1	37.9
	p-value	p = 0.1262	p<.0001	p<.0001	p<.0001
Mixed model ANOVA controlling for cell IDs	WT est.	53.0	38.0	45.0	42.6
	KO est.	53.7	35.4	51.5	49.7
	p-value	p = 0.2094	p<.0001	p<.0001	p<.0001
Mixed model ANOVA, rank-transformed data	WT est.	37.8	28.9	34.9	32.7
	KO est.	38.3	27.7	38.6	37.5
	p-value	p = 0.0744	p<.0001	p<.0001	p<.0001
Nested ANOVA controlling for all experimental blocks	WT est.	51.6	38.9	45.1	43.2
	KO est.	52.8	36.7	51.4	49.5
	p-value	p = 0.0333	p<.0001	p<.0001	p<.0001
Nested ANOVA, rank-transformed data	WT est.	37.0	29.1	34.6	33.1
	KO est.	37.8	28.2	36.0	37.5
	p-value	p = 0.0144	p<.0001	p<.0001	p<.0001

Migration angle for wild-type (WT) vs. PKCƟ-/-, CARMA1-/-, and CCR7-/-, and suB (control) vs. PTX-treated T cells. All angle estimates are in degrees.

### Control for dye effects: use of the factorial ANOVA

In order to compare WT and knockout populations of T cells within the same lymph node, we used the commonly used CFSE and CMTMR dye combination. Because of the potential effect of different dyes on motility, we reversed cell-type and dye combinations between observations to balance the numbers of experiments performed with each dye. However, perfectly balancing observation of the various combinations of cell type with dye is next to impossible as there is no guarantee that the exact same number of cells will appear in the field each day, nor that cell tracks will all be equally long on one day or another. For example, while we made an effort to balance our observations of cell type and dye, due to chance differences in the success of individual observations and the numbers of cells observed in each experiment, among the 122,293 observations (individual time step for a single cell track), for WT:PKCθ-/- experiments, we have 11,940 steps from 543 tracks WT/CFSE, 49,717 steps from 1613 tracks WT/CMTMR, 57,751 steps from 1797 tracks PKCθ-/-/CFSE, and 12,230 steps from 505 tracks PKCθ-/-/CMTMR observations. The only way to guarantee that samples are perfectly balanced is to take the smallest sample size in any cell type/dye observation and throw out observations at random from the other groups until each group is exactly the same size. This would unnecessarily reduce statistical power by eliminating data from the analysis, and still does not account for the possibility that dyes have a biological effect on motility, leading to a source of unexplained variance that increases within-group variance and reduces our power to detect between-group differences [27].

**Fig 3:**
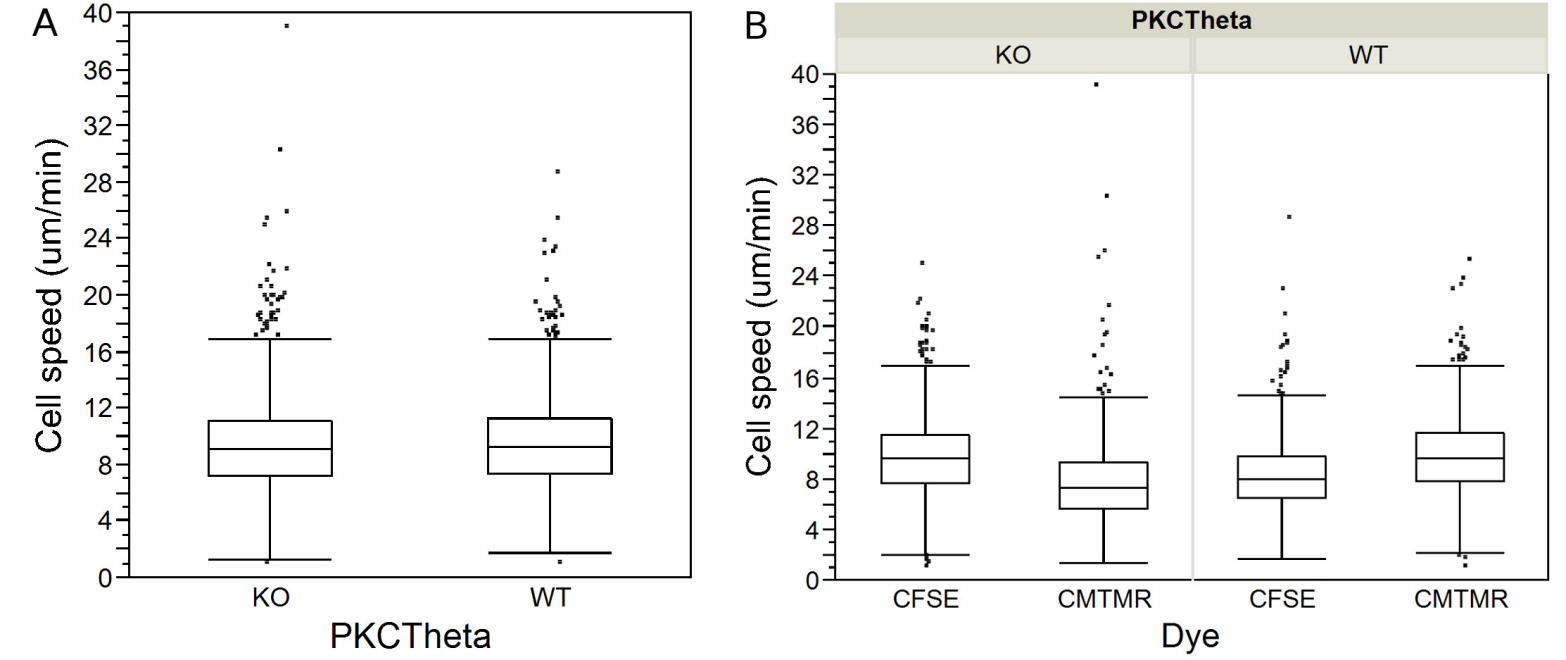
Cell speeds in WT and PKCθ-/- T cells in mouse lymph nodes. Box-plots showing step-based cell speeds calculated from 2P microscopic observation of PKCθ-/- (KO) and wild-type (WT) T cells: A) Cell speeds for KO and WT T cells without regard to dyes used in observations; B) The same data broken out by cell type and dye and the two dyes used to image them, CFSE and CMTMR, illustrating the confounding effect of the dyes on T cell speeds.

**Fig 4:**
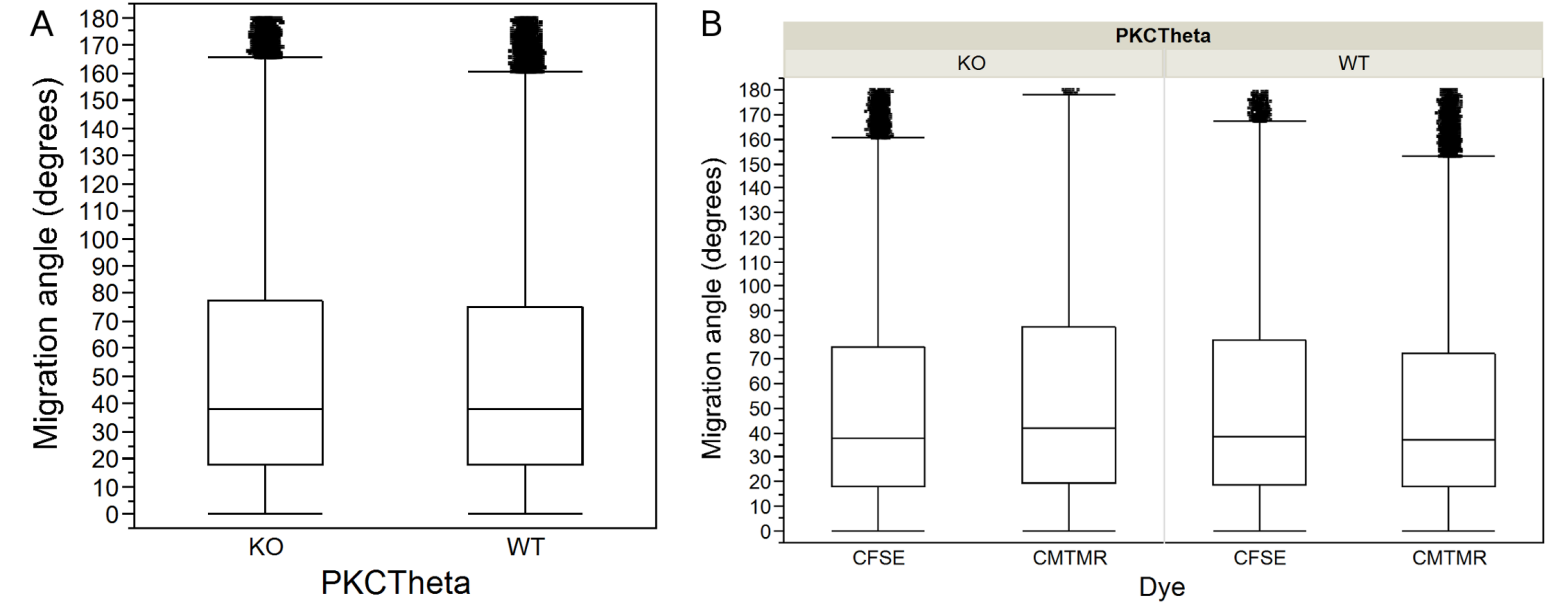
T cell migration angle in WT and PKCθ-/- motility in mouse lymph nodes. Box-plots showing step-based migration angles calculated from 2P microscopic observation of PKCθ-/- (KO) and wild-type (WT) T cells: A) Migration angles for KO and WT T cells without regard to dyes used in observations; B) The same data broken out by cell type and the two dyes used to image them, CFSE and CMTMR, illustrating the confounding effect of the dyes on T cell migration angles.

**Fig 5:**
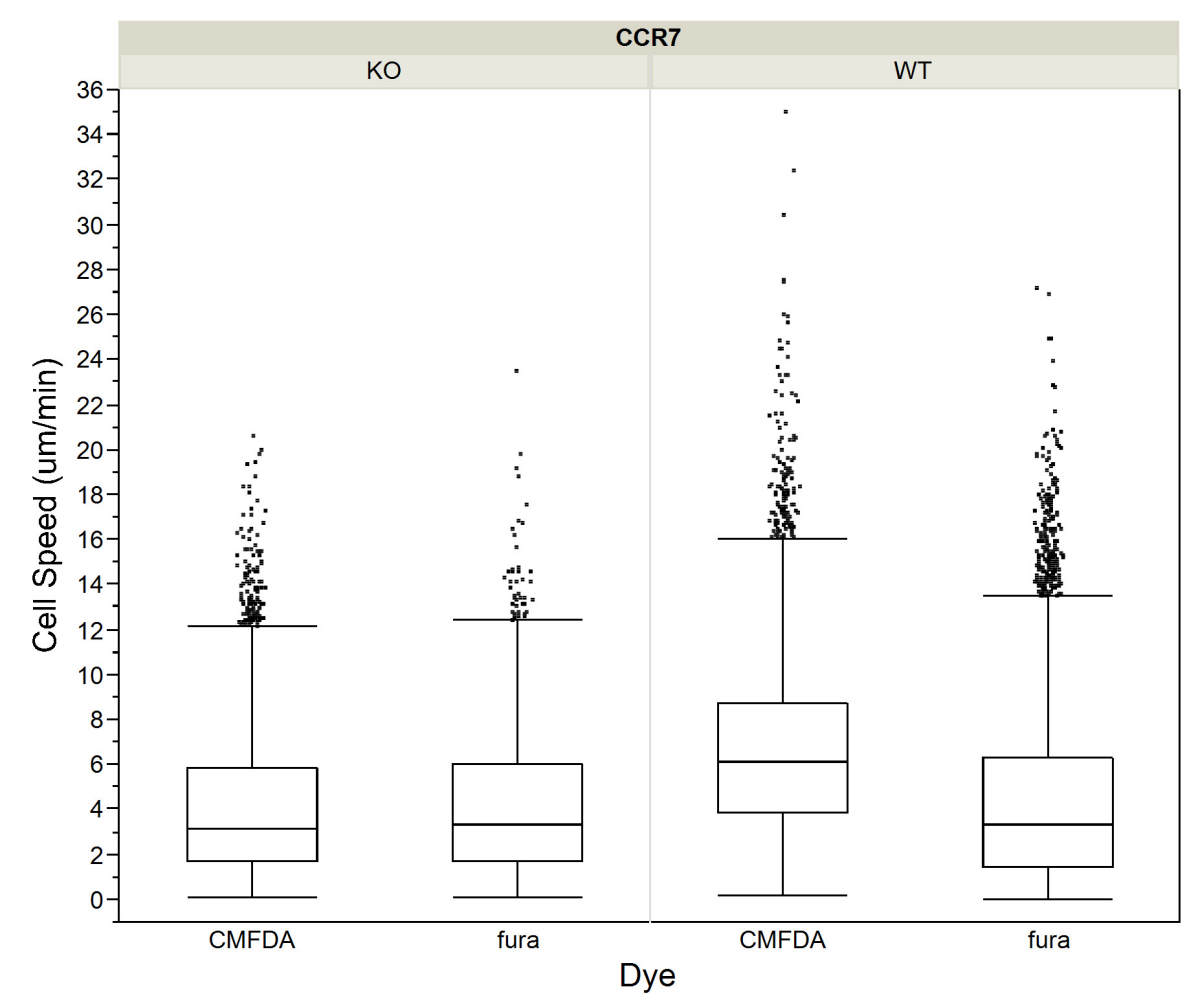
Cell speeds in WT and CCR7-/- T cell motility in mouse lymph nodes, by cell type and dye. Box-plots showing step-based cell speeds calculated from 2P microscopic observation of CCR7-/- (KO) and wild-type (WT) T cells broken out by cell type and the two dyes used to image them, CMFDA and fura.

To account for potential dye effects and differences in the sample size between dyes, we used a two-way or factorial ANOVA. We entered the main effect of the dyes (i.e. the effect of one dye causing cells in general to move faster or slower or to turn more or less than the other); and the dye X cell type interaction (i.e. the effect of one dye making one cell type move faster, and the other slower; or more generally, that dyes will affect different cell types differently) on cell speeds and migration angles. We found a significant effect of the dyes on T cell speed (Table 3) and turning angle (Table 4) in all four data sets we tested. Furthermore, we found that the dyes have a confounding effect on the speed (Table 3) and migration angles (Table 4) of the two cell types. If we simply average the total WT CFSE/WT CMTMR and PKCθ-/- CFSE/PKCθ-/- CMTMR and perform a Mann-Whitney U test (Table 1), we see a statistically significant difference between WT speed at 5.28μm/min and PKCθ-/- at 5.19μm/min (Fig 3A). However, if we break out the WT CFSE—WT CMTMR—PKCθ-/- CFSE—PKCθ-/- CMTMR, we see that the effect of the dye on individual populations was not consistent (Fig 3B). WT cells dyed with CMTMR moved faster than WT cells dyed with CFSE, while the opposite was true for PKCθ-/- T cells (Fig 3B). Thus, the p-value reported by the Mann-Whitney U test for differences in speed for WT versus PKCθ-/- T cells is unreliable because of dependence in the data caused by the use of the CFSE dye in some observations and CMTMR in others. Using the factorial ANOVA to control for the effects of the dyes, our new estimates are 5.05μm/min for WT cells and 4.85μm/min for PKCθ-/-. We did the same analysis for migration angles, and found that with factorial ANOVA, the difference between the turning angles taken by WT (38.5) and PKCθ-/- (38.9) is now not significant, with p = 0.1262 (Fig 4B; and see Table 2). These results show that the significant difference previously found using the Mann Whitney U test was due to the confounding effect of the dye. We found the same dye effects for two other dyes used to assess WT:CCR7-/-, showing that dye effects are likely to be a generalized effect (Fig 5).

**Table 3:** Relative effects of cell populations, dyes, and cell population X dye interactions on cell speed.

	WT:PKCƟ-/-	WT:CARMA1-/-	WT:CCR7-/-	SuB:PTX
Cell population	KO: -0.13***	KO: +0.11***	KO: -0.64***	PTX: -1.06***
Dye	CFSE: +0.08***	CFSE:-.37***	CMFDA: +0.58***	CFSE: -0.05***
Cell type X dye	KO X CFSE: +0.50***	KO X CFSE: -1.11***	KO X CMFDA: -0.59***	PTX X CFSE: +0.23***

Effect sizes for cell population and dye main effects, and the cell population X dye interaction effect on cell speed. Value for knock-out (KO) indicates reduction or increase in cell speed relative to wild-type cells. Dye effect is reduction or increase relative to the other fluorescent dye used in each experiment (CMTMR for PKCƟ, CARMA1, and PTX experiments; fura for CCR7 experiments). Interaction effect is the additional change in speed for the KO and dye combinations. All units are μm/min.

*** p < 0.0001.

**Table 4:** Relative effects of cell populations, dyes, and cell population X (crossed with) dye interactions on migration angle.

	WT:PKCƟ-/-	WT:CARMA1-/-	WT:CCR7-/-	SuB:PTX
Cell population	KO: +0.14	KO: -1.18***	KO: +3.85***	PTX: +2.58***
Dye	CFSE: +0.62***	CFSE: -1.05***	CMFDA: -1.88***	CFSE: +0.01
Cell type X dye	KO X CFSE: -2.81***	KO X CFSE: +3.86***	KO X CMFDA: +1.66***	PTX X CFSE: -3.32***

Effect sizes for cell population and dye main effects, and the cell population X dye interaction effect on migration angle. Value for knock-out (KO) indicates reduction or increase in migration angle relative to wild-type cells. Dye effect is reduction or increase relative to the other fluorescent dye used in each experiment (CMTMR for PKCƟ, CARMA1, and PTX experiments; fura for CCR7 experiments). Interaction effect is the additional change in speed for the KO and dye combinations. All units are degrees.

*** p < 0.0001.

In most cases the magnitude of the dye effect is similar to or larger than that of the experimental treatment. For example, in examining cell speeds: the effect of PKCθ-/- is -0.13μm/min (e.g WT T cells moved 0.13μm/min faster than PKCθ-/- T cells), while the main effect of the dye is 0.08μm/min, and the dye X cell type interaction is 0.50μm/min (the CFSE dye causes a 0.50μm/min increase in PKC-/- cells) (Table 3). We found a similar result for turning angles (Table 4). In fact, only PTX vs. suB cell speeds show that the magnitude of the experimental treatment is substantially larger than both the main and interaction effects of the dyes. These results demonstrate that the dye effects can be large and significant, and if not correctly accounted for statistically, may contribute to effects erroneously attributed to cell type differences.

As noted above for the t-test and Mann-Whitney U test, we find that when we apply this test to rank-transformed data to correct for non-normality, we obtain lower estimates for both speed (e.g. 6.21μm/min for WT cells in un-transformed data from our PKCθ sample vs. 5.05μm/min estimated from rank-transformed data) and turning angles (54.0 degrees vs. 38.5 degrees). Again this reflects the fact that estimates obtained by analysis of un-transformed data correspond to the mean value whereas estimates obtained by analysis of rank-transformed data correspond to the median. However, these estimates still have yet to account for dependence resulting from the fact that steps originate from T cell tracks.

### Incorporating cell-based and step-based analysis

The choice of cell-based vs step-based analysis can influence the estimated differences between the speed and turning angles of WT and PKCθ-/-, CARMA-/-, CCR7-/-, or PTX treated T cells. While it is intuitive to think about the behavior of individual cells, step-based analysis offers the advantage of greater statistical power [28]. Both cell-based and step-based analyses introduce biases into statistical analysis. Step-based analysis doesn’t take into account differences in behavior of individual tracks, and therefore loses an important level of variation by only considering differences in motility parameters pooled across all tracks without regard to the fact that multiple measurements are taken from individuals tracks. Statistical tests assume that there are no correlations (dependence) in the data that are not accounted for in the statistical model. Therefore tests such as the t-test that fail to take into account the important dependence among multiple observations of the same cell are not only less than optimal, but violate this assumption and are therefore inappropriate to apply to step-based data. Cell-based analyses compare track means for these parameters, which may differ due to real differences in the motion of different cells, but without regard to sample size or other information about the cells (e.g. standard deviation). Fast-moving or highly diffusive cells tend to move quickly through the frame, while slow-moving cells or cells with undirected movement may linger in the frame longer, and may therefore be over-represented in step-based data [9]. This is demonstrated by S4 Fig, showing 37 step-based samples of WT speeds, and 82 samples of PKCθ-/- speeds. Using a t-test, we find that the mean speed for the selected WT cells is 1.79μm/min, whereas the mean for KO cells is 9.51μm/min, a large and statistically significant difference (p < 0.001.) However, these two samples represent observations of a single WT cell and a single PKCθ-/- cell, and thus cannot answer the question of whether the *populations* of WT and PKCθ-/- T cells behave differently. While this is an extreme example, it illustrates the exaggeration of our power to test our hypotheses when we group together step-based data without regard to the individual cells that produced the observations. On the other hand, if we convert the data to cell-based parameters we reduce our sample size to two, and in the process we have discarded all other information about our sample, including standard deviation, standard error, and sample size within the steps of a cell.

To resolve the step-based and cell-based conundrum, we again use the factorial ANOVA to enter unique cell IDs as a predictor variable in our model. This allows our model to take into account the fact the multiple observations were made of each cell, and the fact that cells may show individual variation. We analyzed cell speed and migration angle data using mixed-model ANOVA, including the dyes and the dye X cell type interaction we described previously, and also cell IDs to control the dependence in our data owing to repeated observations of the same cells. After including control for cell IDs as a random effect, estimates of cell speed (Table 1) are generally higher and migration angle (Table 2) generally lower than without the cell ID as control. This results from correcting for the over-sampling of cells with slow speeds or high migration angles that linger in the frame longer than cells that move quickly. In fact, for the WT versus CARMA1-/- comparison, a simple Mann-Whitney U test showed a statistically significant difference in cell speed with WT at 7.8μm/min and CARMA1-/- at 7.2μm/min. After controlling for dye effects and the cell ID, our parameters and statistical model are now in effect simultaneously “step-based” and “cell-based”. This more accurate model shows that in fact, CARMA1-/- T cells move slightly faster than WT T cells (WT: 8.4μm/min; CARMA1-/- 9.0μm/min).

### Nested ANOVA controlling for all experimental factors

While we have controlled for dye effects and determined the effect of the cell type (WT versus knockout) on T cell motility, it is possible that other unknown experimental variables may affect our estimates of T cell speed and turning angle. We perform experiments on multiple days, using multiple animals, multiple lymph nodes from different animals, and multiple fields within each lymph node (see Fig 1, and S1, S2 and S3 Figs for experimental setup). Each of these nuisance factors are additional potential sources of dependence that we can check and control for statistically. We do this by entering each factor into our model as random effects much as we do cell ID above. These additional random effects allow for the possibility that observations on particular days, or on particular mice may vary for reasons unrelated to the experimental treatment we are investigating, despite our efforts at methodological control across observations. We again used factorial ANOVA and included the date as our top level random effect and entered the other effects as random effects nested within date, and within each other to match the hierarchical structure in our data (see Fig 1).

We performed our full nested model of cell speeds and migration angles and found that in general, nested analysis does not produce very different estimates or p-values than our mixed model ANOVA controlling for cell IDs (Tables 1 and 2). The 95% confidence intervals for the estimates of date, mouse, lymph nodes, and fields all span zero, indicating that these are non-significant effects in the model. The only random effect we entered with an estimate significantly different from zero is the track IDs (95% CI: 81.1 to 96.0 degrees). These analyses gives us confidence that our experimental observations are well controlled across each field, lymph node, mouse, and date, and that our results are not confounded by differences among these factors.

In one analysis, inclusion of nested factors did allow us to find significant effects where we found non-significant or marginally significant effects in the mixed model controlling for cell IDs. After controlling for dye effects and cell ID, we found a non-significant effect of PKCθ on T cell migration angle in un-transformed data and a marginally significant effect (WT: median migration angle 37.8 degrees; KO: 38.3 degrees; p = 0.0744; see Table 2) in rank-transformed data. When including nested factors in the analysis, we now find significant effects in both un-transformed (WT: 51.6 degrees; KO: 52.8 degrees; p = 0.0333) and rank-transformed data (WT: 37.0 degrees; KO: 37.8 degrees; p = 0.0144). Inclusion of nested factors controlling for experimental blocks can provide greater resolution on differences between experimental groups, even when these factors fail to reach significance because of small sample size (small number of fields, and increasingly smaller numbers of higher level factors up to the days on which experiments were carried out). Agreement between the results of analysis of un-transformed and rank-transformed data gives confidence that these results are not due to violation of the assumption of normality. The median values provided by analysis of rank-transformed data may be regarded as more meaningful as mean values are of questionable usefulness in skewed data such as these.

Interestingly, we also find the surprising result that CARMA1-/- T cells move faster than WT cells that we observed after controlling for dye effects persists when including controls for all nested experimental blocks. Nested analysis of rank-transformed data estimates median speed of 7.8μm/min for WT T cells and 8.4μm/min for KO T cells, with p = 0.0304 (Table 2). Our new statistical analysis approach shows that inappropriate statistics may lead to the incorrect conclusion that CARMA1 deficiency leads to slower motility (if t-test is used). Instead, we find that absence of CARMA1 leads to higher motility, suggesting that CARMA1 normally slows T cell movement in LNs.

## Discussion

Remarkable advances in video microscopy over the last several decades have provided stunning images of cell form and movement in living tissues. Acquisition of this data is expensive in terms of funding invested in the equipment and the time to carry out the observations. With improvements in the statistical techniques used to analyze this valuable data, immunologists can avoid known pitfalls in data analysis and gain substantially more power to obtain accurate estimates of cell motility.

In our study, we resolve three problems in the analysis of *in vivo* T cell motility, and we show how resolving these problems changes quantitative estimates, and in some cases qualitative understanding, of T cell motility. First, we demonstrate how simple rank transformation of data resolves inaccurate estimates that come from using the Student’s t-test due to the non-normally distributed motility data. Second, we show that cell dyes have significant and unpredictable effects on T cell motility, and we demonstrate the use of statistical methods to correctly control for dye effects. Finally, we demonstrate how to leverage the accuracy of cell-based analysis without sacrificing the statistical power of step-based analysis of speed and turning angles.

Researchers in the field have recognized the difficulties of working with complex quantitative motility data, including the effects of the dyes used in two-photon microscopy on lymphocyte motility [13], and the difficulties and potential biases inherent in the choice between cell-based and step-based parameters [9]. We examined in detail the effects of dyes on T cell motility in four data sets using two different dye combinations. In each data set we found not only effects of certain dyes making cells move more slowly, but we also found interaction effects, where dyes may unpredictably affect the motility of T cells of one cell population differently than those of another population. While the effects of the dyes can be lessened through methodological controls, experimental methods cannot fully control for dye effects. (Our nested models including all nested models lost the significance of the dye interaction effects, although their magnitudes were not reduced. This is likely due to small sample sizes for higher level nested effects such as date and mouse.) We show that factorial ANOVA statistical analyses can statistically control the possible confounding effect of the dyes. An additional advantage of the use of the factorial ANOVA is that we can make use of all available data even if the populations are not perfectly balanced. As it is impossible to predict the potential effect of different dyes used to label T cells, this statistical level of control is critical to avoid erroneously reporting an effect of cell type or other experimental treatment that is in fact only due to the choice of dyes.

The techniques we suggest here do not reduce the need for methodological control in experiments. While dye effects are particularly significant, when we use a nested analysis to test for other experimental variables, including field visualized, lymph node, and date of experiment, we find no significant effect of these other experimental variables. These results show that the effect of the dye is a real biological effect, and thus require statistical control in addition to experimental controls. This is in contrast with other experimental variables that can be adequately controlled for by standard experimental techniques.

We also find that WT cells behave differently under different conditions, as shown by the WT motility parameters obtained in the four different sets of results WT:PKCθ-/- (WT speed: 5.8μm/min); WT:CARMA1-/- (WT speed: 7.8μm/min); and WT:CCR7-/- (WT speed 4.92μm/min)(see Table 1). The differences are statistically significant, and are likely due to the fact that each of these sets of data were collected using different microscopes under different experimental conditions. Thus, while we can directly compare WT and knockout T cells within the same sets of experiments done using similar environmental conditions, different experimental setups, (including different microscopes, temperature settings, optics etc.), can affect the precise measure of motility.

We also present a method to resolve the dilemma between step-based and cell-based parameters. However, the mixed modeling we use does not resolve all the potential biases. Fast moving cells may change their behavior after moving out of the field, while slow moving cells are over sampled. While experimental limitations remain, our analysis more completely accounts for the behavior of individual cells in analysis of step-based data, as well as the data structure inherent in these experimental designs. Proper statistical analysis is particularly important when comparing populations of T cells with subtle differences in motility, when cell population effects may be swamped by noise, e.g. that introduced by the fluorescent dyes. However, with these more powerful techniques we may be able to find significant differences between the motility of populations of cells where in fact the magnitudes of those differences are relatively small. For example in our final nested analysis of rank-transformed PKCθ-/- vs. WT T cell speeds, we find a 5% reduction in cell speed, yet the result is highly significant with p<0.0001; and a 1.8% increase in migration angle, with p = 0.0144. We also observe that CARMA1-/- T cells move approximately 8% faster than WT T cells. While the precise biological significance of small decrease or increase in motility is beyond the scope of this study, with the recent increase in the use of computational modeling, we will gain further insight into the effect of both PKCθ and CARMA1 by better estimates of motility. In such cases researchers must consider the biological importance of their results beyond looking for a significant p-value.

Quantitative measurements of T cell motility have been increasingly used as inputs into computational models to better understand T cell behavior [29,30]. Computational modeling requires precise quantitative measures of speed, turning angle, and other parameters to accurately predict a wide variety of immune response parameters, including the initiation a T cell response [7,29,30], generation of CD8 T cell memory [31], CD8 T cell killing [32]. We show that statistical controls can dramatically change the quantitative estimate for the speed and turning angle taken by T cells moving in lymph nodes. The difference between the estimate for T cell speed with and without full statistical control can be more than 10% (Tables 1 and 2), which may lead to significant differences in computational models.

While the statistical techniques we describe here can lead to more precise *quantitative* estimates, we find that different analyses, e.g. parametric vs. non-parametric, factorial ANOVA versus mixed modeling, with control for cell IDs and for experimental variability, produce similar *qualitative* differences between WT and knockout populations. This gives us some assurance that the results we report are not the result of our choice of a particular statistical test. We encourage researchers to check the assumptions of their statistical tests, and if these assumptions are in question, repeat their analyses using alternate methods, including repeating their analysis with rank-transformation in the case of non-normally distributed residuals.

The techniques we have outlined here give researchers more effective tools for addressing widely recognized concerns in analysis of lymphocyte motility: the non-parametric nature of motility data; the effects of fluorescent dyes on lymphocyte motility; and answer the problem of choosing between cell-based and step-based parameters. We have already used these techniques to analyze the effect of PKCθ on T cell motility [20]. These techniques provide a general approach to designing statistical tests that fit the structure of T cell motility data and all possible sources of variance to give the clearest view of T cell motility in vivo.

## Supporting Information

S1 Fig Experimental design and resulting data structure for WT vs CARMA1-/- experiments. Data on CARMA1-/- (KO) and wild-type (WT) T cell motility were collected during experiments on 6 days, using 10 total mice, from which 17 total lymph nodes were extracted, with observation in 22 total microscopic fields, in which 3,883 total tracks were observed, containing 128,611 total step observations.
(TIF)

S2 Fig Experimental design and resulting data structure for WT vs CCR7-/- experiments. Data on CCR7-/- (KO) and wild-type (WT) T cell motility were collected during experiments on 2 days, using 2 total mice, from which 7 total lymph nodes were extracted, with observation in 8 total microscopic fields, in which 1,402 total tracks were observed, containing 15,931 total step observations.
(TIF)

S3 Fig Experimental design and resulting data structure for suB- vs PTX-treated T cell experiments. Data on subunit B- (suB, control) and PTX-treated T cell motility were collected during experiments on 7 days, using 10 total mice, from which 17 total lymph nodes were extracted, with observation in 22 total microscopic fields, in which 4,096 total tracks were observed, containing 111,251 total step observations.
(TIF)

S4 Fig Step-based data on cell speeds for a single PKCθ-/- and single WT T cell. Plot of step-based cell speeds calculated from 2P microscopic observation of a single PKCθ-/- (KO) and a single wild-type (WT) T cells. A t-test would incorrectly conclude that KO cells move at faster speeds than WT (p < 0.001). In fact, these data points represent samples of the motility of only one KO and one WT cell. The t-test does not take into account the dependence among these observations. We do not have sufficient data in this sample to conclude anything about differences between WT and KO cell populations when the identity of the individual cells from which these observations were made are taken into account.
(TIFF)

S5 Fig Model specification of nested model in JMP. Specification of the final nested model for analysis of PKCθ-/- vs wild-type T cell speed. The model includes factors: PKCθ (KO or WT), dye, and the cell-type X dye interaction; and hierarchically nested factors, date, mouse, lymph node, field, and cell, each entered into the model as random effects. See http://www.jmp.com/support/help/Construct_Model_Effects.shtml for further information on nested factors and model specification in JMP. See http://stmc.health.unm.edu/tools-and-data/ for replication data and JMP procedure for the nested model.
(TIF)
