# Supplementary figures and images for: Bringing Statistics Up to Speed with Data in Analysis of Lymphocyte Motility
Source: PLoS One. 2015 May 14;10(5):e0126333. doi: 10.1371/journal.pone.0126333 (PMC4431811; doi:10.1371/journal.pone.0126333)

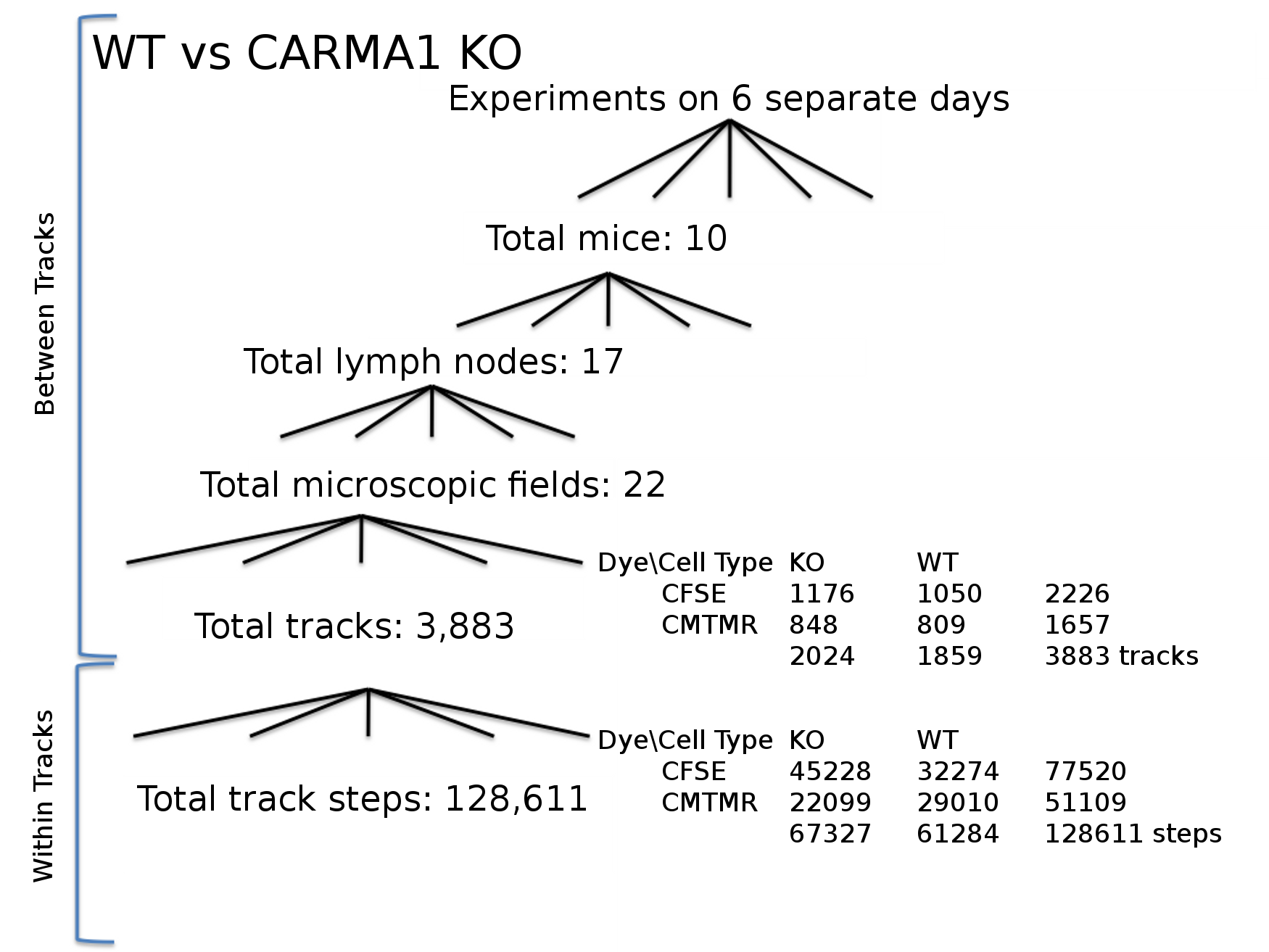

Supplement: S1 Fig — Data on CARMA1-/- (KO) and wild-type (WT) T cell motility were collected during experiments on 6 days, using 10 total mice, from which 17 total lymph nodes were extracted, with observation in 22 total microscopic fields, in which 3,883 total tracks were observed, containing 128,611 total step observations. (TIF) [file pone.0126333.s001.tif]

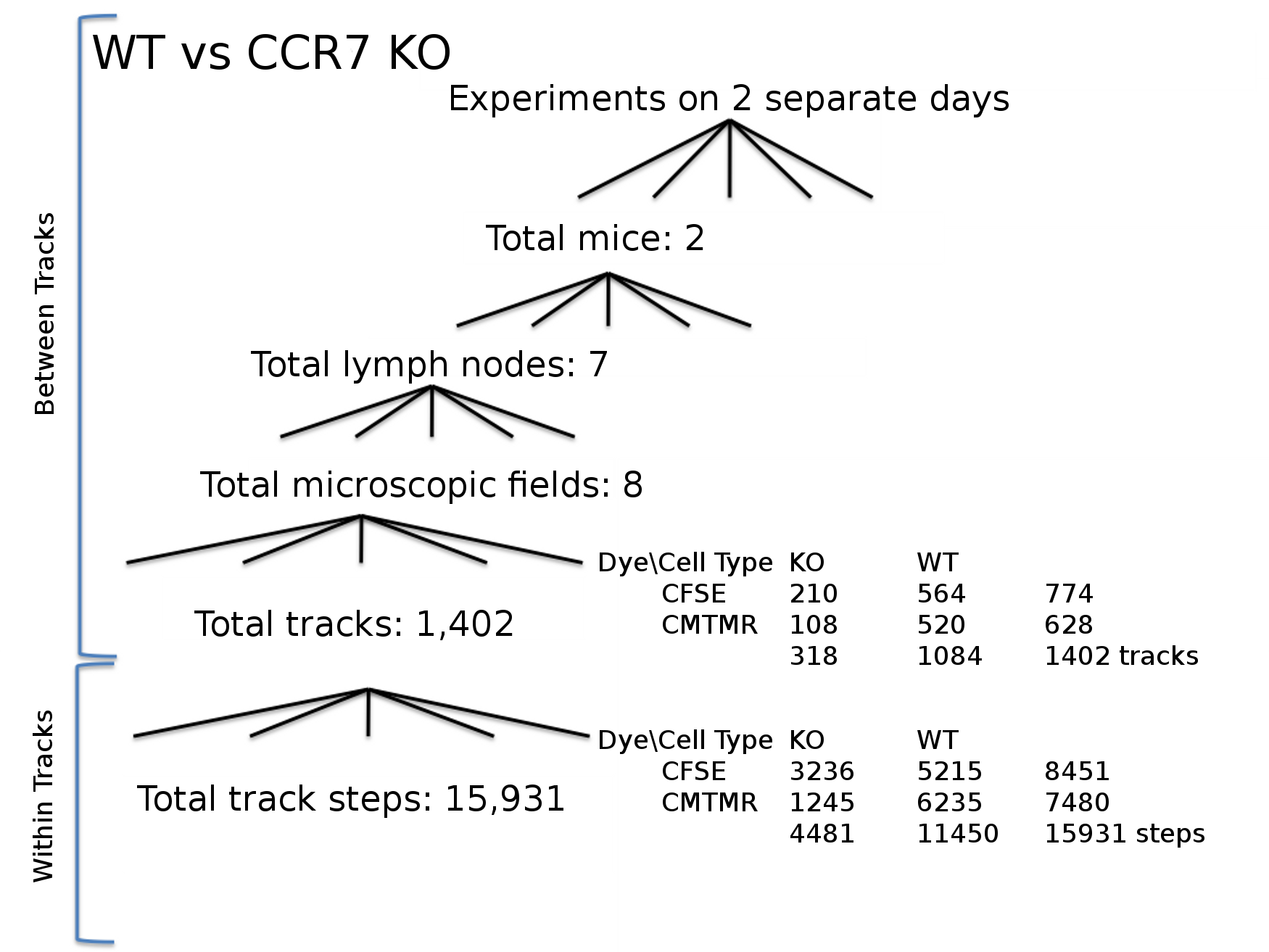

Supplement: S2 Fig — Data on CCR7-/- (KO) and wild-type (WT) T cell motility were collected during experiments on 2 days, using 2 total mice, from which 7 total lymph nodes were extracted, with observation in 8 total microscopic fields, in which 1,402 total tracks were observed, containing 15,931 total step observations. (TIF) [file pone.0126333.s002.tif]

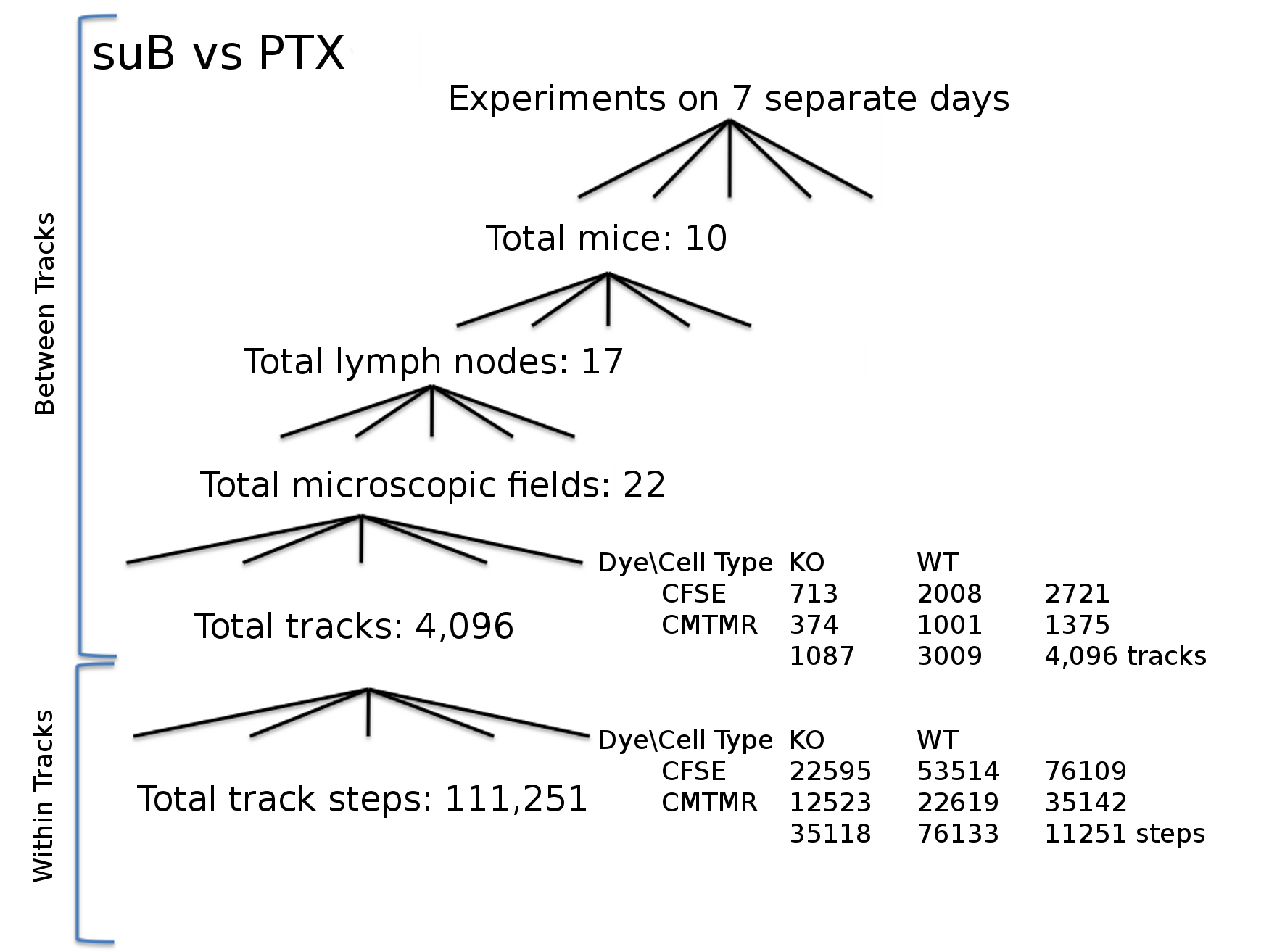

Supplement: S3 Fig — Data on subunit B- (suB, control) and PTX-treated T cell motility were collected during experiments on 7 days, using 10 total mice, from which 17 total lymph nodes were extracted, with observation in 22 total microscopic fields, in which 4,096 total tracks were observed, containing 111,251 total step observations. (TIF) [file pone.0126333.s003.tif]

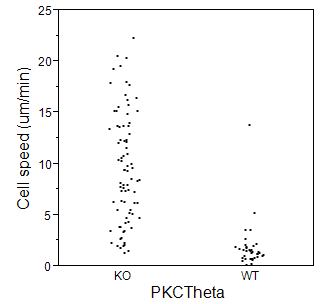

Supplement: S4 Fig — Plot of step-based cell speeds calculated from 2P microscopic observation of a single PKCθ-/- (KO) and a single wild-type (WT) T cells. A t-test would incorrectly conclude that KO cells move at faster speeds than WT (p < 0.001). In fact, these data points represent samples of the motility of only one KO and one WT cell. The t-test does not take into account the dependence among these observations. We do not have sufficient data in this sample to conclude anything about differences between WT and KO cell populations when the identity of the individual cells from which these observations were made are taken into account. (TIFF) [file pone.0126333.s004.tiff]

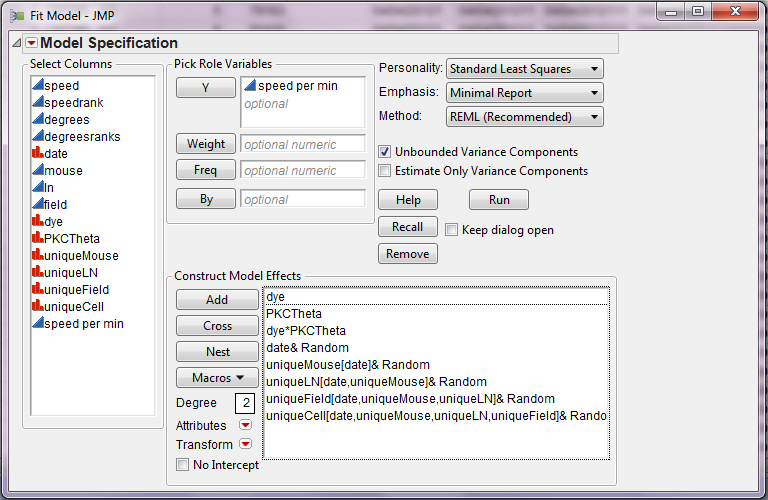

Supplement: S5 Fig — Specification of the final nested model for analysis of PKCθ-/- vs wild-type T cell speed. The model includes factors: PKCθ (KO or WT), dye, and the cell-type X dye interaction; and hierarchically nested factors, date, mouse, lymph node, field, and cell, each entered into the model as random effects. See http://www.jmp.com/support/help/Construct_Model_Effects.shtml for further information on nested factors and model specification in JMP. See http://stmc.health.unm.edu/tools-and-data/ for replication data and JMP procedure for the nested model. (TIF) [file pone.0126333.s005.tif]
